# Uncovering the Chemical Composition and Biological Potentials of *Bupleurum lancifolium* Hornem. from Jordan

**DOI:** 10.3390/molecules29122730

**Published:** 2024-06-08

**Authors:** Nezar Al-Bataineh, Sultan T. Abu-Orabi, Suhair B. Shannag, Hala I. Al-Jaber, Tareq T. Bataineh, Wael A. Al-Zereini, Mahmoud A. Al-Qudah

**Affiliations:** 1 College of Pharmacy, Al Ain University of Science and Technology, Abu Dhabi 112612, United Arab Emirates; 2 Department of Medical Analysis, Faculty of Science, Tishk International University, Erbil 44001, Iraq; sultan.abuorabi@tiu.edu.iq; 3 Department of Chemistry, Faculty of Science, Yarmouk University, P.O. Box 566, Irbid 21163, Jordan; 2018104020@ses.yu.edu.jo (S.B.S.); tariq.b@yu.edu.jo (T.T.B.); 4 Department of Chemistry, Faculty of Science, Al-Balqa Applied University, Al-Salt 19117, Jordan; hala.aljaber@bau.edu.jo; 5 Department of Biological Sciences, Faculty of Science, Mutah University, Al-Karak 61710, Jordan; wzereini@mutah.edu.jo

**Keywords:** *Bupleurum lancifolium*, essential oil, antioxidants activity, AChE inhibition, cytotoxicity, quercetin derivatives

## Abstract

The current study was designed to uncover the chemistry and bioactivity potentials of *Bupleurum lancifolium* growing wild in Jordan. In this context, the fresh aerial parts obtained from the plant material were subjected to hydrodistillation followed by GC/MS analysis. The main components of the HDEO were γ-patchoulene (23.79%), β-dihydro agarofuran (23.50%), α-guaiene (14.11%), and valencene (13.28%). Moreover, the crude thanolic extract was partitioned to afford two main major fractions, the aqueous methanol (BLM) and butanol (BLB). Phytochemical investigation of both fractions, using conventional chromatographic techniques followed by careful inspection of the spectral data for the isolated compounds (NMR, IR, and UV-Vis), resulted in the characterization of five known compounds, including α-spinasteryl (**M1**), ethyl arachidate (**M2**), ethyl myristate (**M3**), quercetin-3-*O*-β-d-glucopyranosyl-(1-4”)-α-L-rhamnopyranosyl (**B1**), and isorhamnetin-3-*O*-β-d-glucopyranosyl-(1-4”)-α-L-rhamnopyranosyl (**B2**). The TPC, TFC, and antioxidant activity testing of both fractions and HDEO revealed an interesting ABTS scavenging potential of the BLB fraction compared to the employed positive controls, which is in total agreement with its high TP and TF contents. Cytotoxic evaluation tests revealed that BLM had interesting cytotoxic effects on the normal breast cell line MDA-MB-231 (ATCC–HTB-26) and the normal dermal fibroblast (ATCC^®^ PCS-201-012) and normal African green monkey kidney Vero (ATCC-CCL-81) cell lines. Despite both the BLB and BLM fractions showing interesting AChE inhibition activities (IC_50_ = 217.9 ± 5.3 µg/mL and 139.1 ± 5.6 µg/mL, respectively), the HDEO revealed an interestingly high AChE inhibition power (43.8 ± 2.7 µg/mL) that far exceeds the one observed for galanthamine (91.4 ± 5.2 µg/mL). The HDEO, BLM, and BLB exhbitied no interesting antimicrobial activity against *Bacillus cereus*, *Bacillus subtilis*, *Staphylococcus aureus*, *Escherichia coli*, or *Pseudomonas aeruginosa*.

## 1. Introduction

Umbelliferae (Apiaceae) family, famously known as the carrot family, is the 16th largest family of flowering plants, with more than 3540 species belonging to 446 genera [1,2]. Aromatic flowering plants belonging to this family include economically important foods and spices, like caraway, anise, fennel, coriander, cumin, carrot, and many others. Most umbelliferae plants are annual, biennial, or perennial herbs, and less frequently shrubs or trees, that are known to be distributed worldwide but mostly in temperate climates [1]. Among the different species belonging to this plant family, about 250 species have been reported as medicinally significant apiaceous plants utilized for ages in traditional medicine for treating an assortment of illnesses [3].

The *Bupleurum* genus is a genera belonging to the Umbelliferae family, comprising about 180–190 species, mostly reported to grow wild in Eurasia, North Africa, and the Mediterranean [2]. In Jordan, there are five species belonging to this genus, including *Bupleurum brevicaule* Schltdl., *Bupleurum Gerardi* All., *Bupleurum lancifolium* Hornem., *Bupleurum nodiflorum* Sm., and *Bupleurum semicompositum* L. [4].

Several *Bupleurum* species, either alone or in combination with additional components, have been included in many pharmaceutical preparations and prescribed for the treatment of different ailments, including the common cold [5], inflammation [6], hepatitis [7], cancer [8], and fever associated with malaria [8]. Additionally, *Bupleurum* species have been used as analgesics to alleviate persistent abdominal pain in the hypochondriac area of the chest, to prevent amenorrhea, and to treat and protect against chronic hepatitis, nephrotic syndrome, and autoimmune illnesses [9]. Moreover, other uses include the treatment of diabetes, vertigo, vomiting, dry throat, and cholecystitis, as well as the enhancement of wound healing and deafness [10]. The high concentration of polyacetylenes, which have very neurotoxic effects, is thought to be the cause of the poisonous nature of some of this genus’s dangerous species [11].

Phytochemical investigation of several *Bupleurum* species has resulted in the identification of several classes of secondary metabolites, including flavonoids, coumarins, lignans, triterpenes saikosaponins, and polyacetylenes [12], in addition to the occurrence of free fatty acids, like pinellic acid, angelic acid, petrosylic acid, and lignoceric acid [13]. These bioactive metabolites can operate as key substances in the therapy or avoidance of several severe diseases [14,15].

**Figure 1:**
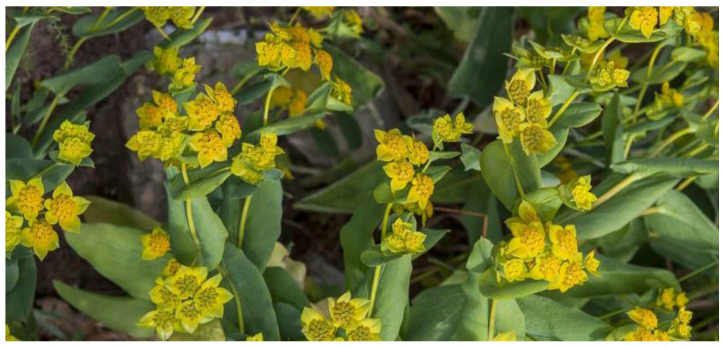
*Bupleurum lancifolium*.

*Bupleurum lancifolium* Hornem. is an annual plant; the flowers are bisexual, with yellowish simple leaves having long and slender shapes (Figure 1). The plant has been reported to grow wild in many countries and regions, like Algeria, Cyprus, Egypt, Greece, Iran, Iraq, Kuwait, Lebanon–Syria, Libya, Madeira, Morocco, Palestine, Sinai, Spain, Tunisia, Turkey, Turkmenistan, and Western Sahara. The plant is reported to have anti-inflammatory, antitussive, hepatoprotective, anti-ulcer and immunomodulatory, antispasmodic, diaphoretic, antioxidant, and antimicrobial activities [16]. Previous phytochemical investigations on the leaves of *B. lancifolium* resulted in the isolation of two triterpenoid saponins (3-*O*-[α-l-rhamnopyranosyl (1→4)-β-d-glucopyranosyl] echinocystic acid 28-*O*-β-d-glucopyranosyl ester and 3-O-[α-L-rhamnopyranosyl (1→4)-β-d-glucopyranosyl] oleanolic acid 28-*O*-β-d-glucopyranosyl ester) and two other flavonoids, including isorhamnetin 3-rutinoside and rutin [17]. In Turkey, Saraçoğlu et al. (2012) investigated the chemical composition of the essential oil obtained from different organs of several *Bupleurum* species [18]. The results revealed spathulenol and α-pinene as the primary constituents of the oil obtained from the flowers. Hexacosane and pentacosane dominated the essential oil obtained from fruit oil, while the roots oil contained mainly hexadecanoic acid and heptacosane [18]. Another study reported the fatty acid composition of the oils extracted from *B. lancifolium* collected from the central Anatolia region of Turkey [19], reporting oleic acid, linoleic acid, and linolenic acids as the main components of the oils [19]. Moreover, hexane extract obtained from *B. lancifolium* leaves and seeds extract contained ω-3, ω-6, palmitic acid, and γ-linolenic acid and is considered an important source of ω-3 and ω-6 compounds among several *Bupleurum* species [20].

The main goal of the present study was to uncover the chemistry of *B. lancifolium* through investigating the composition of its hydrodistilled essential oil (HDEO) and aqueous methanol (BLM) and butanol (BLB) fractions. Furthermore, the two fractions (BLM and BLB) were assayed for their total phenolic contents (TPCs) and total flavonoid contents (TFCs). Both fractions, along with the HDEO, were tested for their antioxidant activity (DPPH and ABTS), antibacterial potentials (against a set of Gram-positive and Gram-negative bacteria), cytotoxic activity, and neuroprotective effects.

## 2. Results and Discussion

### 2.1. Chemical Constituents of the Hydrodistilled Essential Oil (HDEO)

The chemical composition of the HDEO obtained from fresh aerial parts of *B. lancifolium* was analyzed by GC-MS, and the results are shown in Table 1, while Figure S1 displays the obtained GC/MS chromatogram. A total of 48 different components, amounting to 99.76% of the total composition, were identified in this HDEO.

**Figure 2:**
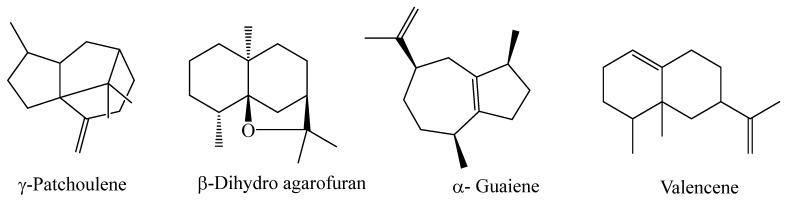
Main components detected in the HDEO of *B. Lancifolium*.

**Figure 3:**
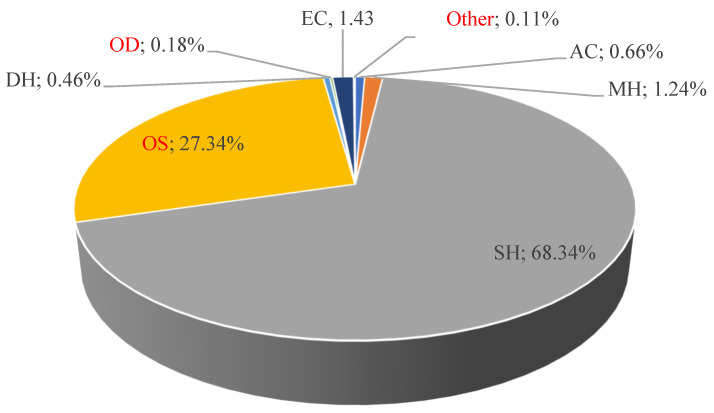
Chemical groups of essential oil. Monoterpene hydrocarbons (MHs); sesquiterpene hydrocarbons (SHs); oxygenated sesquiterpenes (OS); aliphatic compounds (ACs); diterpene hydrocarbons (DHs); oxygenated diterpenes (ODs); ester and carboxylic acid (EC); and other compounds.

As can be deduced from Table 1, the HDEO obtained from fresh aerial parts of *B. lancifolium* contained mainly γ-patchoulene (23.79%), β-dihydro agarofuran (23.50%), α- guaiene (14.11%), and valencene (13.28%); the structures of these constituents are shown in Figure 2. The main classes of compounds detected in this oil are shown in Figure 3.

Comparing our current results with those of previous investigations reveals some qualitative and quantitative differences. The essential oil obtained from Turkish *B. lancifolium* contained mainly oleic acid ω9 (69.36%), linoleic acid ω6 (13.88%), and palmitic acid (10.29%). Other components were detected in lower amounts, such as myristoleic acid ω5 (1.25%), palmitoleic acid ω7 (0.90%), and heptadecanoic acid ω8 (0.02%) [18]. The essential oil obtained from the leaves and seeds of *B. lancifolium* from Iran contained mainly linolenic acid ω-3 (17.1 and 48.1%, respectively), linoleic acid ω-6 (14.1 and 22.2%), palmitic acid (25.7 and 10.1%), and γ-linolenic acid (21.3 and 8.2%). Other components were detected in lower amounts, such as myristic acid (0.7 and 2.3%), stearic acid (3.1 and 4.8%), and arachidic acid (0.3 and 2.5%) [20].

#### Chemical Constituents of the BLM and BLB Fractions from *B. lancifolium*

A phytochemical investigation using conventional chromatographic separation of *B. lancifolium*’s main fractions resulted in the isolation and identification of five known compounds, three from the BLM (**M1**–**M3**) fraction and two from the BLB fraction (**B1** and **B2**). With careful inspection of the spectral data for the isolated compounds (IR, NMR, UV-Vis, and HRESIMS, Figures S2–S20), these compounds are identified as α-spinasteryl (**M1**), ethyl arachidate (**M2**), ethyl myristate (**M3**), quercetin-3-*O*-β-d-glucopyranosyl-(1-4”)-α-l-rhamnopyranosyl (**B1**), and isorhamnetin-3-*O*-β-d-glucopyranosyl-(1-4”)-α-L-rhamnopyranosyl (**B2**). The structures of the isolated compounds are shown in Figure 4. 

### 2.2. Total Flavonoid (TFC) and Phenol (TPC) Contents and Antioxidant Activity

The results of the TPC, TFC, and antioxidant activity determined for the two fractions (BLB and BLM) are shown in Table 2. The obtained data clearly indicate that BLB had the highest TPC and TFC (849.46 ± 4.37 mg/g gallic acid equivalents; 405.23 ± 1.47 mg/g quercetin equivalents) compared to the BLM fraction. The DPPH^•^ and ABTS^•+^ scavenging activity of the crude extracts and essential oil were dose dependent and increased with an increasing concentration (Figure S21. As shown in Table 2, both the BLB and BLM fractions showed good DPPH^•^ radical scavenging powers ((IC_50_ = 8.0 ± 0.79 and 11.3 ± 0.20 μg/mL, respectively), which were less than that observed for HDEO (45.4 ± 0.50 μg/mL) when compared with the two positive controls ascorbic acid and α-tocopherol (1.58 ± 0.035 and 1.79 ± 0.01 μg/mL, respectively). The BLB fraction had the highest ABTS^•+^ scavenging potential (10.0 ± 0.54 μg/mL) compared to the BLM fraction (18.0 ± 0.35 μg/mL) and HDEO (41.0 ± 0.70 μg/mL). The observed activity for both fractions could be attributed to their TP and TF contents [21,22]. Despite the fact that no phenolic compounds were detected in the GC/MS analysis of the HDEO, the moderate antioxidant potential recorded for the essential oil could be attributed to its terpenoidal content. The main components detected in the HDEO, including γ-patchoulene, 7-epi-α-selinene, β-dihydro agarofuran, and α-guaiene, are known for their antioxidant properties [23,24].

### 2.3. Antibacterial, AChE Inhibition, and Cytotoxic Activity Assays

The antibacterial effects of the HDEO and the BLM and BLB fractions were evaluated against *Bacillus cereus*, *Bacillus subtilis*, *Staphylococcus aureus*, *Escherichia coli,* and *Pseudomonas aeruginosa*. The tested fractions and EO showed no antibacterial potentials at a 2 mg/mL concentration level and, accordingly, were considered inactive against all tested bacterial strains. However, the BLM fraction showed potent cytotoxic activity against the MDA-MB-231 breast cancer cell line at concentrations lower than that effective in inhibiting proliferation of both the normal fibroblast and Vero cell lines with the IC_50_ (44.7 ± 1.3, 50.8 ± 2.1, and 106.9 ± 2.5 µg/mL, respectively) (Table 3). It is noteworthy that, although both the BLB and HDEO were ineffective against the tested cell lines, both extracts, as well as the BLM, revealed an ability to inhibit the activity of the AChE enzyme and, thereby, have potential roles in improving the cholinergic synapsis. 

The anti-AChE activities of the BLB, BLM, and HDEO were concentration dependent. Interestingly, the BLB and BLM displayed moderately weak AChE inhibition powers when compared to the positive control galantamine (IC_50_ = 217.9 ± 5.3 and 139.1 ± 5.6 vs. 6.4 ± 2.1 µg/mL, respectively). The HDEO showed a stronger AChE inhibition potency compared to the two other fractions (IC_50_ = 43.8 ± 2.7 µg/mL), which was three- to five-fold more effective than the BLB and BLM (Table 4). 

The present study’s findings coincide with the literature on the medical applications of different *Bupleurum* species [5,6,7,15,16,18,19,20]. The inactivities of the tested BLM, BLB, and HDEO toward all tested bacterial strains have been previously reported by several studies for some *Bupleurum* species [18], including *B. lancifolium*; its essential oil [19] and crude extract [25] were devoid of activities against both Gram-negative and -positive bacteria. Intriguingly, the cytotoxic and anticholinergic activities of the BLM crude extract could be attributed to its contents of fatty acids and sterols (i.e., α-spinasteryl). Their activities were attributed to their lipophilic characteristic, which acts directly on biomembranes and affects membrane permeability, deteriorates plasma membrane, and might dissipate mitochondrial membrane potential [16,26,27]. 

Alpha-spinasterol has demonstrated potent cytotoxic activity on human ovarian, cervical Hela, and colon CACO-2 cell lines [28,29]. Furthermore, this compound was also reported to cause inhibition in the growth of breast MDA-MB-231 and MCF-7 and ovarian SKOV-3 cell lines, acting mainly as an anti-estrogenic compound and possibly exerting its effect by binding to ER receptors and, thus, causing ER^+^ MCF-7 cells to be more sensitive to α-spinasterol, caused by overexpression of p53 and downregulation in the cell cycle control Cdk4, leading to G0–G1 cell cycle arrest [30]. Moreover, the ability of BLM to antagonize the AChE can also be correlated with the presence of α-spinasterol as a major component in this fraction, which is in total agreement with a previous report on its AChE inhibitory effect (IC_50_ = 44.19 ± 2.59 µg/mL) [31].

However, the documented anticholinergic activity of HDEO herein could be attributed to its high sesquiterpenes content. The β-dihydro agarofuran derivatives obtained from the seeds of *Maytenus disticha* and *M. magellanica* inhibited AChE (IC_50_ = 17.0 ± 0.016 and 740.0 ± 0.045 µM, respectively) [32]. β-Dihydro agarofuran derivatives in *Chilean Celastraceae* extract caused an interesting AChE inhibitory effect (IC_50_ values ranging from 120 ± 0.003 to 740.0 ± 0.035 µM) [33]. The activity of this compound is attributed to its ability to form hydrogen bonds with the peripheral anionic site (PSA) at the entrance of the enzyme. Furthermore, an α-guaiene-rich extract obtained from *Xylocarpus moluccensis* roots showed anti-AchE potency (IC_50_ = 21 µg/mL) [34]. On the other hand, the presence of flavonoid glycosides like quercetin-3-*O*-β-d-glucopyranosyl-(1-4”)-α-l-rhamnopyranosyl and isorhamnetin-3-*O*-β-d-glucopyranosyl-(1-4”)-α-l-rhamnopyranosyl), as well as isorhamnetin derivatives, could account for the observed moderate AChE inhibitory effects of BLB fraction. Previously, isorhamnetin was reported to cause instability in Aβ aggregate neuroblastoma SH-SY5Y cells [27], and enhance synaptic plasticity and neurogenesis in the prefrontal cortex and hippocampus in scopolamine-induced amnesia mice model by improving the level of brain-derived neurotrophic factor (BDNF) [35]. Moreover, the interaction between the active site of the AChE enzyme and the flavonol skeleton of both the quercetin and isorhamnetin derivatives decreases the activity of AChE [36], with the quercetin derivative being more potent than the isorhamnetin analogs.

## 3. Materials and Methods

### 3.1. General

^1^H-NMR spectra were recorded on a Bruker Avance III 500 MHz spectrometer with TMS as an internal standard. ^13^C-NMR spectra were recorded at 125 MHz. High-resolution mass spectra (HRESIMS) were acquired by electrospray ionization with the positive-mode technique using a Bruker APEX-4 Mass spectrometer. UV-Vis spectra were recorded on a Shimadzu UV-1800 UV/Visible Scanning Spectrophotometer. TLC was performed on silica gel 60 GF254 precoated glass plates (0.25 or 0.50 mm in thickness, Macherey-Nagel). The compounds were visualized under UV light or spraying with sulfuric acid–anisaldehyde spraying reagent followed by heating. Analysis of the HDEO’s constituents was performed on an Agilent 6890 series II—5973 GC-MS spectrometer interfaced with an HP chemstation.

### 3.2. Plant Material

*B. lancifolium* was collected during the full flowering phase (April-2019) in the Zahar region (32.568737°, 35.793078°). The plant material’s identity was certified by Prof. Dr. Jamil N. Lahham (Department of Biology, Faculty of Science, Yarmouk University, Irbid, Jordan). A reference specimen (BL/Ap/2019) was stored in the natural products laboratory—Prof. Mahmoud A. Al-Qudah, Yarmouk University, Irbid, Jordan.

### 3.3. Hydrodistillation of Essential Oil

Fresh aerial parts of *B. lancifolium* (200 g) were minced and suspended in distilled water (150 mL) and then subjected to hydrodistillation with a Clevenger apparatus for 3 h [37,38]. The obtained oil was separated by extraction with diethyl ether (2.0 mL) twice. After evaporation of the diethyl ether, the resulting oil was dissolved in GC-grade *n*-hexane, dried over anhydrous sodium sulfate, and then stored in amber glass vials at 4–6 ºC.

The identification of the separated essential oil components was achieved by comparing their calculated Kovats retention (KI) to (C_8_–C_20_) *n*-alkanes values with a column of identical polarity and under the same chromatographic conditions, as well as matching their recorded mass spectra with those listed in the built-in libraries’ spectra (NIST, Gaithersburg, MD, USA and Wiley Co., Hoboken, NJ, USA). The principal components of the extracts were further identified by injecting authentic standard reference compounds under the same chromatographic conditions and from the literature [39].

### 3.4. Extraction and Isolation

The plant material was dried and crushed to a fine powder (8.0 kg) and then defatted for 10 days at room temperature using petroleum ether (20 L). Then, the defatted plant material was extracted with ethanol at room temperature (5 times, 7 days each). The combined ethanolic extract was concentrated under reduced pressure by evaporation, and the resulting alcoholic residue (700 g) was partitioned according to the procedure described in the literature [40] to obtain the aqueous methanol (BLM; 73.3 g) and butanol (BLB; 184.73 g) fractions.

The aqueous methanol fraction (BLM; 73.3 g) was adsorbed on 100.91 g of mesh silica gel and then chromatographed in a column (45 × 6 cm, 500 g mesh silica gel) packed in hexane and eluted with a gradient hexane/ethyl acetate mixture of increasing polarity A total of 177 fractions were collected (250 mL each) and then consolidated into 6 main subfractions (BLM-I–BLM-VI) based on their TLC behavior. Isolation and purification from these collective subfractions were then achieved by a combination of CC and TLC utilizing proper solvent systems. Three compounds (**M1**, **M2**, and **M3**) were isolated from the BLM fraction.

Similarly, the butanol fraction (BLB; 184.73 g) was adsorbed on 200.42 g mesh silica gel and then subjected to chromatography in a column (40 × 7 cm, 800 g mesh silica) packed in chloroform (CHCl_3_) and eluted with a gradient mixture of CHCl_3_:MeOH of increasing polarity. A total of 213 fractions (250 mL each) were collected and then grouped into 5 major subfractions (BLB-I–BLB-V) according to their TLC behavior. Isolation and purification from these collective subfractions were then achieved by a combination of CC and TLC or a suitable solvent. This whole process resulted in the isolation and characterization of **2** compounds of the butanol fraction.

- α-Spinasteryl (**M1**)

Fraction BLM-II-2 offered a white solid after being washed with methanol. Compound **M1** was identified as α-spinasteryl on the basis of a careful inspection of its spectral data (Figures S2–S4) [41]. IR (KBr) *ν* (cm^−1^): 3439 (OH), 1459(C=C). *R_f_* = 0.70 (40% MeOH/CF) and 0.42 (20% EtOAc/hex). ^1^H–NMR (CDCl_3_) *δ* ppm: 3.63 (1H, m, H-3), 5.04 (1H, m, H-22), 5.16 (1H, dd, *J* = 8.64, 15.12, H-23), and 5.18 (1H, dd, *J* = 8.64, 15.12, H-7). ^13^C–NMR (CDCl_3_) δ ppm: methyls: (12.06, C-18), (12.27, C-29), (19.00, C-19), (21.11, C-27), (21.39, C-26), and (23.02, C-21); methenes: 21.55 (C-11), 25.41 (C-15), 29.64 (C-16), 29.71 (C-6), 31.48 (C-2), 31.88 (C-25), 37.14 (C-1), 37.99 (C-12), and 40.85 (C-4); methine carbons: 28.53 (C28)), 39.46 (C-20), 40.26 (C-5), 49.44 (C-9), 51.26 (C-24), 55.13 (C17), 55.89 (C-14), 71.07 (C-3), 117.47 (C7), 129.43 (C23), 138.19 (C-22), and 138.19 (C-22); quaternary carbons: 34.22 (C-10), 29.64 (C-16),43.29 (C-13), and 139.58 (C-8). HRESIMS *m/z* = 413.37614 [M + H]^+^ (calcd. for [C_29_H_48_O]^+^: 412.3705).

- Ethyl arachidate (**M2**)

A solid was collected from the fraction BLM-II-3. This solid was purified with distilled methanol multiple times to obtain a pure white precipitate that was identified as ethyl arachidate based on its spectral data (Figures S5–S8) [41].
IR (KBr) ν (cm^−1^): 1717 (C=O group), 1113–1288 (C–O group), 1472 (CH_2_ bending), and 2917 and 2849 (C–H stretching). *R_f_* = 0.43 (10% EtOAc/hex) and 0.14 (100 % CF). ^1^H-NMR (CDCl_3_) *δ* ppm: 0.88 (3H, t, *J* = 6.52, 7.04 Hz, H-20, and H-2’), 1.43 (32H, m, H-4-H19), 1.59 (2H, m, H-3), 2.27 (2H, m, H-2), 4.01 (2H, m, H1’). ^13^C-NMR (CDCl_3_) *δ* ppm: methyls: 14.15 (C-20, C-2’); methenes: 25.04–31.94 (C-3-C-19), 34.44 (C-2), 64.42 (OCH_2_, C-1’), and 174.08 (C=O, C-1). EI-MS: M^+^ (*m/z*) 340.3128 (C_22_H_44_O_2_).

- Ethyl myristate (**M3**)

Compound **M3** was obtained from the fraction BLMII-1. The obtained solid was purified upon washing several times with distilled methanol, affording ethyl myristate as a pure white solid. (Figures S9–S12) [42,43].
IR (KBr) *ν* (cm^−1^): 1966 (C=O group), 1116–1246 (C–O group), 1463 (CH_2_ bending), and 2917 and 2848 (C–H stretching). *R_f_* = 0.5 (10% EtOAc/hex.) and 0.65 (100% CF). ^1^H-NMR (CDCl_3_) *δ* ppm: 0.88 (3H, t, *J* = 6.64Hz, H-14), 1.25 (20H, br s, H4-H13), 1.61 (2H, m, H-3), 2.33 (2H, t, *J* = 8.64 Hz, H-2), and 4.11 (2H, q, *J* = 7.12, H-1’). ^13^C-NMR (CDCl_3_) δ (ppm): 14.13 (CH_3_, C-14), 14.25 (CH_2_, C-2), 22.70 (CH_2_, C-13), 29.07–32.77 (CH_2_, C-4-C12), 25.73 (CH, C-3), 34.33 (CH_2_, C-2), 60.21(OCH_2_, C-1’), and 174.0 (C=O, C-1). EI-MS M^+^ (*m/z*): 256.2317 (C_16_H_32_O_2_).

- Quercetin-3-*O*-β-d-glucopyranosyl-(1-4”)-α-L-rhamnopyranosyl (**B1**)

Treatment of the subfraction BLB-III-4 with distilled methanol resulted in the precipitation of a pure light-yellow-colored solid. Careful investigation of the spectral data led to the identification of this compound as quercetin-3-*O*-β-d-glucopyranosyl-(1-4”)-α-L-rhamnopyranosyl (Figures S13–S16) [44].
IR (KBr) *ν* (cm^−1^): 1657 (C=O), 3425 (OH), and 1573, 1505 (aromatic nucleus). *R_f_* = 0.43 (40% MeOH/CF) and 0.1 (30% EtOAc/hexane). ^1^H-NMR (DMSO-d_6_) *δ* ppm: 6.19 (1H, d, *J* = 1.96, H-6), 6.38 (1H, d, *J* = 1.96, H-8), 6.85 (1H, d, *J* = 8.2, H-5’), and 7.55 (1H, d, *J* = 2.2, H-6’); 1st sugar moiety: 3.04 (1H, m, H-4“), 3.22 (1H, m, H-3”), 3.28 (1H, m, H-2”), 3.30 (1H, m, H-5”), and 3.71 (1H, d, *J* = 10.2 H-6”); 5.35 (1H, d, *J* = 7.4, H-1”); 2nd sugar mioety: 0.99 (3H, d, *J* = 6.2 Hz, H-6‴), 3.07 (1H, M, H-4‴), 3.25 (2H, m, H-2‴,5‴), 3.39 (1H, m, H-3‴), and 4.38 (1H, bs, 5.6, H-1‴); ^13^C-NMR (DMSO-d_6_) *δ* ppm: 93.6 (C-8), 98.7 (C-6), 103.9 (C-10), 115.2 (C-2’), 116.2 (C-5’), 121.1 (C-6’), 121.5 (C-1’), 133.3 (C-3), 144.7 (C-4’), 148.4 (C-3’), 156.4 (C-2), 156.5 (C-9), 161.2 (C-5), 164.2 (C-7), and 177.3 (C-4); 1st sugar moiety: 66.9 (C-6”), 70.0 (C-4”), 74.0 (C-2”), 75.9 (C-5”), 76.40 (C-3”), and 101.2 (C-1”); 2nd sugar moiety: 17.7 (C-6‴), 68.2 (C-5‴), 70.3 (C-2‴), 70.5 (C-3‴), 71.8 (C-4‴), and 100.7 (C-1‴). UV/Vis: UV λ_max_ (MeOH) nm: 359.51 (band I) and 268.62 (band II); +NaOMe: 402.53 (band I) and 272.52 (band II); +AlCl_3_: 430.76 (Band Ia), 302 (Band IIa), and 272.52 (Band IIb); +HCl: 393.95 (Band Ia), 301 (Band IIa), and 268.62 (Band IIb). HRESIMS *m/z* = 611.1546 [M + H]^+^ (calcd. for [C_27_H_30_O_16_]^+^: 610.1534).

- Isorhamnetin-3-*O*-β-d-glucopyranosyl-(1-4”)-α-L-rhamnopyranosyl (**B2**)

Treatment of the subfraction BLB-II-2 with distilled methanol resulted in the precipitation of pure light-yellow-colored solid. Careful investigation of the spectral data led the identification of this compound as isorhamnetin-3-*O*-β-d-glucopyranosyl-(1-4”)-α-L-rhamnopyranosyl (Figures S17–S20) [44].
IR (KBr) *ν* (cm^−1^): 1657 (C=O), 3425 (OH groups), 1573, and 1505 (aromatic nucleus). *R_f_* = 0.43 (40%MeOH/CF) and 0.1(30%EtOAc/hexane). ^1^H-NMR (DMSO-d_6_) *δ* ppm: 6.19 (1H, d, *J* = 2.0, H-6), 6.42 (1H, d, *J* = 2.0, H-8), 6.92 (1H, d, *J* = 8.5, H-5’), 7.52 (1H, dd, *J* = 8.5, 2.0, H-6’), and 7.86 (1H, d, *J* = 2.2, H-2’); 1st sugar moiety: 3.04 (1H, m, H-4”), 3.22 (1H, m, H-2”), 3.28 (1H, m, H-3”), 3.27 (1H, m, H-5”), and 3.35 (1H, d, *J* = 10.2 H-6”); 5.43 (1H, d, *J* = 17.8, H-1”); 2nd sugar moiety: 0.98 (3H, d, *J* = 6.2 Hz, H-6‴), 3.04 (1H, M, H-4‴), 3.24 (2H, m, H-5‴), 3.26 (2H, m, H-2‴), 3.32 (1H, m, H-3‴), and 4.41 (1H, d, *J* = 1.0 Hz, H-1‴); ^13^C-NMR (DMSO-d_6_) *δ* ppm: 93.8 (C-8), 98.8 (C-6), 103.9 (C-10), 113.2 (C-2′), 115.2 (C-5′), 122.2 (C-6’), 121.0 (C-1’), 132.9 (C-3), 146.7 (C-4’), 149.4 (C-3’), 156.4 (C-2), 156.5 (C-9), 161.1 (C-5), 164.4 (C-7), and 177.2 (C-4); 1st sugar moiety: 66.8 (C-6”), 70.0 (C-4”), 74.2 (C-2”), 75.9 (C-5”), 76.3 (C-3”), and 101.1 (C-1”); 2nd sugar moiety: 17.7 (C-6‴), 68.3 (C-5‴), 70.3 (C-2‴), 70.5 (C-3‴), 71.7 (C-4‴), and 100.9 (C-1‴). UV/Vis: UV λ_max_ (MeOH) nm: 359.51 (band I) and 268.62 (band II); +NaOMe: 402.53 (band I) and 272.52 (band II); +AlCl_3_: 430.76 (band Ia), 302 (band IIa), and 272.52 (band IIb); +HCl: 393.95 (band Ia), 301 (band IIa), and 268.62 (band IIb). HRESIMS *m/z* = 625.1702 [M + H]^+^ (calcd. for [C_28_H_32_O_16_]^+^: 624.1690).

### 3.5. Determination of Total Flavonoid (TFC) and Phenol (TPC) Contents

Both the BLM and BLB fractions were tested for their TFC using Folin–Ciocalteu assay methods according to the procedure described in [37,38] with slight modification. Briefly, a 1.0 mL aliquot of the stock solution prepared from each fraction/EO (1 mg/mL) was diluted in 4.0 mL distilled water; then, 0.30 mL sodium nitrite solution (5% NaNO_2_, *w*/*v*) was added to a 10.0 mL volumetric flask. After 5 min, 0.30 mL of aluminum chloride solution (10% AlCl_3_, *w*/*v*) was added. The resulting solution was incubated for further 6 min and then 2.0 mL of 1.0 M NaOH solution was added to the mixture. The volume of the final solution was adjusted to 10.0 mL with distilled water. After 15 min, the absorbance was measured at 510 nm. Methanol was used as a blank. The TFC is expressed in mg quercetin/g of dry extract. 

The TPCs for the BLB and BLM fractions were determined by aluminum chloride assay, as described previously [37,38]. Briefly, 0.5 mL aliquot of each fraction stock solution (1 mg/mL) was treated with 2.5 mL of Folin–Ciocalteu reagent (2N) (diluted ten-fold) and 2 mL of Na_2_CO_3_ (75 g/L). The mixture was kept at room temperature for 15 min; then, the absorbance was recorded at 765 nm. Methanol was used as a blank solution. The TPC is reported in mg Gallic acid/g of dry extract.

### 3.6. Determination of Antioxidant Activity

The antioxidant activity of the two fractions (BLB and BLM) and the HDEO was evaluated by the DPPH^•^ and ABTS^•+^ assay methods, as described previously [37,38]. Ascorbic acid and α-tocopherol were used as positive controls (Figure S21). 

### 3.7. Evaluation of Biological Activities

#### 3.7.1. Antibacterial Activity 

The antibacterial activities of the BLB, BLM, and HDEO were determined as described in the literature [45,46], following the Clinical and Laboratory Standards Institute’s guidelines (CLSI, 2012), using the agar diffusion test. Several concentrations of the fractions/EOs (300 µg/disc, 500 µg/disc, 1 mg/disc, and 2 mg/disc) were applied on a surface of 6 mm sterile, blank discs placed on the top of Müller Hinton agar plates containing 10^6^ cells/mL of the tested bacterial strains. The included bacterial strains were Gram-positive (*B. cereus* ATCC 11778; *B. subtilis* ATCC 6633; and *S. aureus* ATCC 43300) and Gram-negative strains (*E. coli* ATCC 25922 and *P. aeruginosa* ATCC 13048). The tests were conducted in three independent experiments. 

#### 3.7.2. In Vitro Cytotoxicity Assay

The antiproliferative potencies of the BLB, BLM and HDEO were evaluated against three monolayer cell lines, namely, the human breast adenocarcinoma MDA-MB-231 (ATCC–HTB-26), the normal dermal fibroblast (ATCC^®^ PCS-201-012), and the normal African green monkey kidney Vero (ATCC-CCL-81) cell lines following a methodology described previously [46]. The cells were treated with different concentrations (50-200 μg/mL) of the tested samples. Daunorubicin hydrochloride (Sigma-Aldrich, Darmstadt- Germany) was used as a positive control, while untreated cells were regarded as the negative control. Data are expressed as the mean ± SD of three independent experiments; the concentration that caused 50% inhibition in cell proliferation was extrapolated from a dose–response curve plotted for percentage inhibitions against respective sample concentrations. 

#### 3.7.3. Acetylcholinesterase (AChE) Inhibition Assay

The potential anti-AChE activities of the BLB, BLM, and HDEO were measured on the basis of Ellman’s method using a 96-well plate, as described previously [46]. The samples were tested at different concentrations (100 and 300 µg/mL) with galantamine used as the positive control. The enzyme inhibition percentage is reported as the mean ± standard deviation (SD for three independent trials). The concentration of the tested sample that caused a 50% inhibition in enzyme activity (IC_50_) was interpolated from a dose–response curve plotted for the percentage activity inhibitions against the respective sample concentrations.

## 4. Conclusions

In this study, the chemical composition of the HDEO obtained from the aerial parts of *B. lancifolium* grown in Jordan was analyzed by GC/MS and is reported here for first time. Oxygenated sesquiterpenes (OS) was the main class of compounds detected in this HDEO. The main components detected were γ-patchoulene, β-dihydro agarofuran, α-guaiene, and valencene. Additionally, phytochemical investigation of the BLM and BLB fractions, using conventional chromatographic techniques followed by careful inspection of the spectral data (NMR, IR, UV-Vis, and EI-MS), resulted in the isolation and characterization of five known compounds, including α-spinasteryl (**M1**), ethyl arachidate (**M2**), ethyl myristate (**M3**), quercetin-3-*O*-β-d-glucopyranosyl-(1-4”)-α-L-rhamnopyranosyl (**B1**), and isorhamnetin-3-*O*-β-d-glucopyranosyl-(1-4”)-α-L-rhamnopyranosyl (**B2**). The two fractions (BLM and BLB) were assayed for their total phenolic contents (TPCs) and total flavonoid contents (TFCs), and then, along with the HDEO, were tested for their antioxidant activities (DPPH and ABTS), antibacterial potentials (against a set of Gram-positive and Gram-negative bacteria), cytotoxic activities, and neuroprotective effects. *B. lancifolium* extracts represent potential agents for treating cancer and neurogenerative diseases.

## Figures and Tables

**Figure 4:**
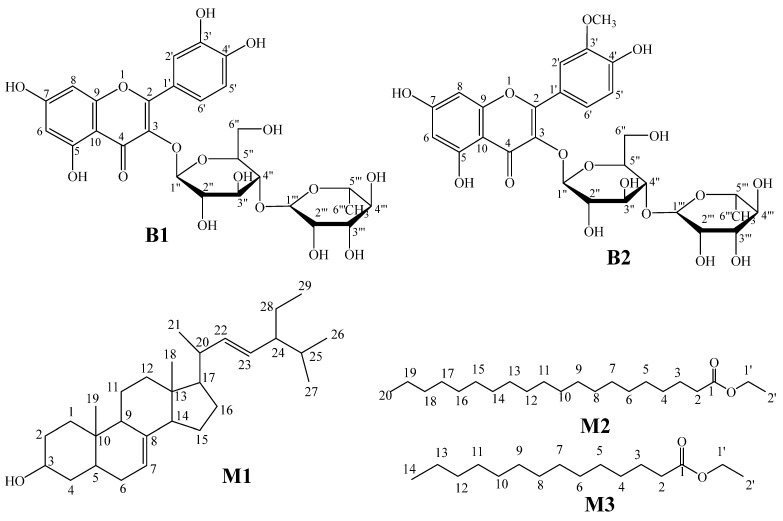
Chemical constituents of *B. lancifolium* isolated from the BLM and BLB fractions.

**Table 1:** Chemical constituents and their percentage of the composition in the HDEO obtained from aerial parts of *B. lancifolium*.

No.	*R_t_*	KI ^a^	KI_Lit._	Compound	Type	% A ^b^	Mode of Identification
1	4.045	800	800	*n*-Octane	AC	0.11	MS ^c^, KI, CoI ^d^
2	5.788	885	895	(4*Z*)-Heptenal	AC	0.19	MS, KI
3	6.869	927	926	Tricyclene	MH	0.37	MS, KI
4	8.132	971	975	Sabinene	MH	0.05	MS, KI
5	8.634	988	979	β-Pinene	MH	0.06	MS, KI
6	10.331	1034	1029	β-Phellandrene	MH	0.76	MS, KI, CoI
7	13.034	110	1100	Undecane	AC	0.16	MS, KI
8	23.32	1335	1338	δ-Elemene	SH	0.29	MS, KI, CoI
9	25.24	1379	1376	α-Copaene	SH	0.45	MS, KI
10	25.582	1387	1388	β-Bourbonene	SH	0.07	MS, KI
11	25.781	1392	1388	β-Cubebene	SH	0.81	MS, KI
12	26.986	1420	1418	Ethyl-(2*E*)-decanoate	EC	0.83	MS, KI
13	27.553	1438	1439	α-Guaiene	SH	14.11	MS, KI, CoI
14	27.708	1438	1441	Aromadendrene	SH	0.64	MS, KI
15	27.911	1443	1444	Guaia-6,9-diene	SH	0.06	MS, KI, CoI
16	28.212	1450	1460	allo-Aromadendrene	SH	0.23	MS, KI
17	28.497	1457	1463	*cis*-Cadina-1(6),4-diene	SH	0.33	MS, KI, CoI
18	28.724	1462	1472	Dauca-5,8-diene	SH	1.47	MS, KI
19	30.086	1495	1496	Valencene	SH	13.28	MS, KI
20	30.358	1502	1503	β-Dihydro agarofuran	OS	23.50	MS, KI
21	30.534	1506	1502	γ-Patchoulene	SH	23.79	MS, KI
22	30.827	1514	1522	7-epi-α-Selinene	SH	8.98	MS, KI
23	30.891	1515	1509	Germacrene A	SH	1.24	MS, KI
24	31.083	1520	1512	δ-Amorphene	SH	0.22	MS, KI
25	31.231	1524	1529	Zonarene	SH	0.46	MS, KI
26	31.437	1529	1523	δ-Cadinene	SH	1.82	MS, KI, CoI
27	31.535	1532	1534	*trans*-Cadina-1,4-diene	SH	0.05	MS, KI
28	31.888	1540	1538	α-Cadinene	SH	0.05	MS, KI
29	32.037	1544	1535	10-epi-Cubebol	OS	0.06	MS, KI
30	33.912	1592	1595	*cis*-dihydro-Mayurone	OS	0.91	MS, KI
31	36.093	1650	1640	*epi*-α-Cadinol	OS	0.08	MS, KI
32	36.193		1643	α-Muurolol	OS	0.25	MS, KI, CoI
33	36.652	1664	1663	7-epi-α-Eudesmol	OS	0.48	MS, KI
34	36.9	1671	1669	(*E*)-10,11-Dihydroatlantone	OS	0.05	MS, KI
35	37.219	1680	1670	14-Hydroxy-9-epi-(*E*)-caryophyllene	OS	0.12	MS, KI
36	38.978	1728	1723	Methyl-tetradecanoate	EC	0.11	MS, KI
37	39.624	1746	1741	Mint sulfide	Other	0.11	MS, KI
38	42.937	1841	1868	(*E*)-β-Santalol acetate	OS	1.45	MS, KI
39	43.134	1846	1860	(*Z*,*Z*)-Farnesyl acetone	OS	0.38	MS, RI
40	44.269	1880	1900	dihydro-Columellarin	OS	0.08	MS, KI
41	44.935	1899	1901	*epi*-Laurenene	DH	0.19	MS, KI
42	45.906	1929	1931	Beyerene	DH	0.27	MS, KI
43	49.684	2046	2036	(*Z*)-Falcarinol	AC	0.11	MS, KI
45	51.178	2092	2085	Methyl linoleate	EC	0.20	MS, KI
46	51.378	2098	2133	Linoleic acid	EC	0.28	MS, KI, CoI
47	57.138	2275	2189	1-Docosene	AC	0.09	MS, KI
48	62.515	2441	2332	Methyl daniellate	OD	0.18	MS, KI
				Total identified		99.76	

^a^ KI = Kovats retention index experimentally calculated. ^b^ % Area of the peak. ^c^ MS: Identification by mass spectrum. ^d^ CoI: Co-injection with authentic compound.

**Table 2:** Results for the TPC (mg/g gallic acid equivalents) and TFC (mg/g quercetin equivalents) of the BLM and BLB fractions and the IC_50_ (μg/mL) of the antioxidant activities of the HDEO and BLM and BLB fractions obtained from *B. lancifolium.*

Extract/Reference	TPC	TFC	IC_50_ (**µg/mL)**	
			DPPH^•^	ABTS^•+^
HDEO	-	-	45.4 ± 0.50	41.0 ± 0.70
BLM	790.76 ± 1.86	150.61 ± 2.50	11.3 ± 0.20	18.0 ± 0.35
BLB	849.46 ± 4.37	405.23 ± 1.47	8.0 ± 0.79	10.0 ± 0.54
Ascorbic acid	-	-	1.58 ± 0.03	1.78 ± 0.06
α-Tocopherol	-	-	1.79 ± 0.01	2.33 ± 0.01

**Table 3:** Cytotoxic effects of extracts (BLB, BLM, and essential oil) and daunorubicin against the MDA-MB-231, fibroblast, and Vero cancer cell lines.

Sample	IC_50_ (µg/mL)		
	MDA MB-231	Fibroblast	Vero
BLM	44.7 ± 1.3 ^a^	50.8 ± 2.1 ^a^	106.9 ± 2.5 ^b^
Daunorubicin	2.3 ± 0.2 *	0.5 ± 0.1 *	6.2 ± 1.7 *

IC_50_ values (µg/mL) with similar letters are not significantly different from each other based on the post hoc Tukey HSD test. Asterisks indicate statistically significant differences in the IC_50_ values of the positive control to the tested samples (* *p* ˂ 0.05).

**Table 4:** Acetylcholine esterase (AChE) enzyme % inhibition activities and IC_50_ (µg/mL) evaluations of the BLB, BLM, and HDEO compared to the positive control galantamine.

Sample	% Inhibition		IC_50_
	µg/mL		
	100	300	
HDEO	66.1 ± 4.1	72.3 ± 7.3	43.8 ± 2.7 ^a^
BLB	21.1 ± 2.0	62.9 ± 4.1	217.9 ± 5.3 ^b^
BLM	39.4 ± 2.4	73.5 ± 3.4	139.1 ± 5.6 ^c^
Galantamine	-	-	6.4 ± 2.1 ^d^

IC_50_ values (µg/mL) with similar letters are not significantly different from each other based on the post hoc Tukey HSD test.

## Data Availability

Data are contained within the article and supplementary materials.

## Supplementary Materials

The following supporting information can be downloaded at: https://www.mdpi.com/article/10.3390/molecules29122730/s1. Figure S1: Gas chromatogram for oil derived by hydro-distillation from aerial portions of B. Lancifolium; Figure S2: IR of compound M1; Figure S3: 1H-NMR of compound M1; Figure S4: 13C-NMR of compound M1; Figure S5: IR of compound M2; Figure S6: Mass of compound M2; Figure S7: 1H-NMR of compound M2; Figure S8: 13C-NMR of compound M2; Figure S9: IR of compound M3; Figure S10: Mass of compound M3; Figure S11: 1H-NMR of compound M3; Figure S12: 13C-NMR of compound M3; Figure S13: IR of compound B1; Figure S14: 1H-NMR of compound B1; Figure S15: 13C-NMR of compound B1; Figure S16: UVI-Vis of compound B1; Figure S17: IR of compound B2; Figure S18: 1H-NMR of compound B2; Figure S19: 13C-NMR of compound B2; Figure S20: UV-Vis of compound B2; Figure S21: DPPH and ABTS radical scavenging activity results for the tested fractions and HDEO.
