# Supplementary material for: Uncovering the Chemical Composition and Biological Potentials of Bupleurum lancifolium Hornem. from Jordan
Source: Molecules. 2024 Jun 8;29(12):2730. doi: 10.3390/molecules29122730 (PMC11206654; doi:10.3390/molecules29122730)
Supplement: Supplementary file 1 [file molecules-29-02730-s001.zip › molecules-3033341-supplementary.pdf]

Supplementary

Uncovering the chemical composition and biological potentials of *Bupleurum Lancifolium* Hornem from Jordan

Nezar Al-Bataineh [1]*, Sultan T. Abu-Orabi [2], Suhair B. Shannag[3], Hala I. Al-Jaber [4], Tareq T. Bataineh [3], Wael A. Al-Zereini[5], Mahmoud A. Al-Qudah [3]*

[1]College of Pharmacy, Al Ain University of Science and Technology, Abu Dhabi, United Arab Emirates.
[2]Department of Medical Analysis, Faculty of Science, Tishk international University, Erbil, KRG, Iraq
[3]Department of Chemistry, Faculty of Science, Yarmouk University, P.O. Box.**566**, Irbid, **21163**, Jordan.
[4]Department of Chemistry, Faculty of Science, Al-Balqa Applied University, Al-Salt, **19117**, Jordan;
[5]Department of Biological Sciences, Faculty of Science, Mutah University, 61710 Al-Karak, Jordan.

Abstract:

The current study was designed to uncover the chemistry and bioactivity potentials of *Bupleurum lancifolium* growing wild in Jordan. In this context, the fresh aerial parts obtained from the plant material were subjected to hydrodistillation followed by GC/MS analysis. The main components of the HDEO were γ-patchoulene (23.79%), β-dihydro agarofuran (23.50%), α- guaiene (14.11%), and valencene (13.28%). Moreover, the crude thanolic extract was partitioned to afford two main major fractions, the aqueous methanol (BLM) and butanol (BLB). Phytochemical investigation of both fractions, using conventional chromatographic techniques followed by careful inspection of the spectral data for the isolated compounds (NMR, IR, UV-Vis and HRESIMS) resulted in the characterization of five known compounds including α-spinasteryl (M1), ethyl arachidate (M2), ethyl myristate (M3), quercetin-3-O-β-D-glucopyranosyl-(1-4'')-α-L-rhamnopyranosyl (B1) and isorhamnetin -3-O-β-D-glucopyranosyl-(1-4'')-α-L-rhamnopyranosyl (B2). TPC, TFC and antioxidant activity testing of both fractions and HDEO revealed interesting ABTS scavenging potential of the BLB fraction as compared to employed positive controls, which was in total agreement with its high TP and TF contents. Cytotoxic evaluation tests revealed that BLM had interesting cytotoxic effects on normal breast cell lines MDA-MB-231 (ATCC–HTB-26), normal dermal fibroblast (ATCC® PCS-201-012) and normal African green monkey kidney Vero (ATCC-CCL-81) cell lines. Despite that both BLB and BLM fractions showed interesting AChE inhibition activities (IC50: 217.9±5.3 μg/mL; 139.1±5.6 μg/mL., respectively), HDEO revealed interestingly high AChE inhibition power (43.8±2.7 μg/mL) that far exceed the one observed for galanthamine (91.4±5.2 μg/mL). The HDEO, BLM and BLM had no interesting antimicrobial activity against Bacillus cereus, Bacillus subtilis, Staphylococcus aureus, Escherichia coli and Pseudomonas aeruginosa.

Keywords: *Bupleurum lancifolium*; Essential oil; Antioxidants activity; AChE inhibition; cytotoxicity, quercetin derivatives

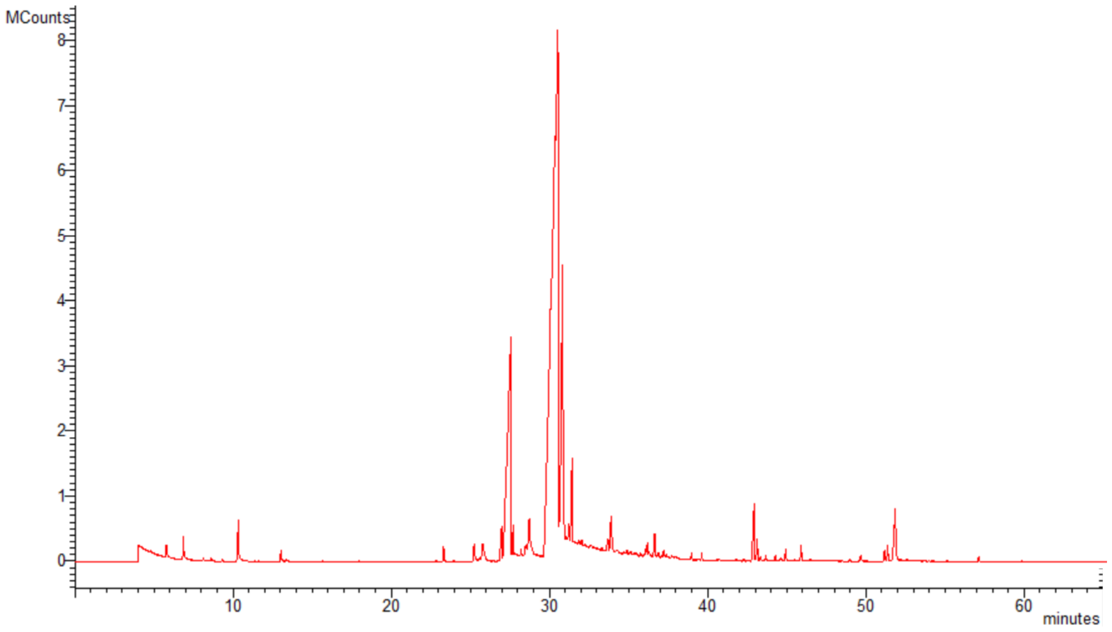

Figure S1: Gas chromatogram for oil derived by hydro-distillation from aerial portions of *B. Lancifolium*.

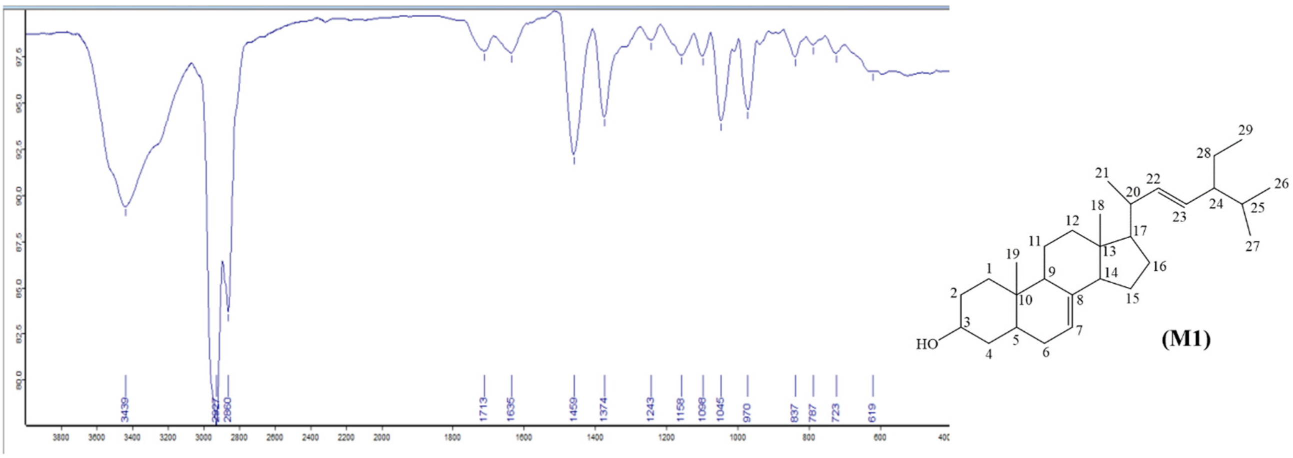

Figure S2: IR of compound M1

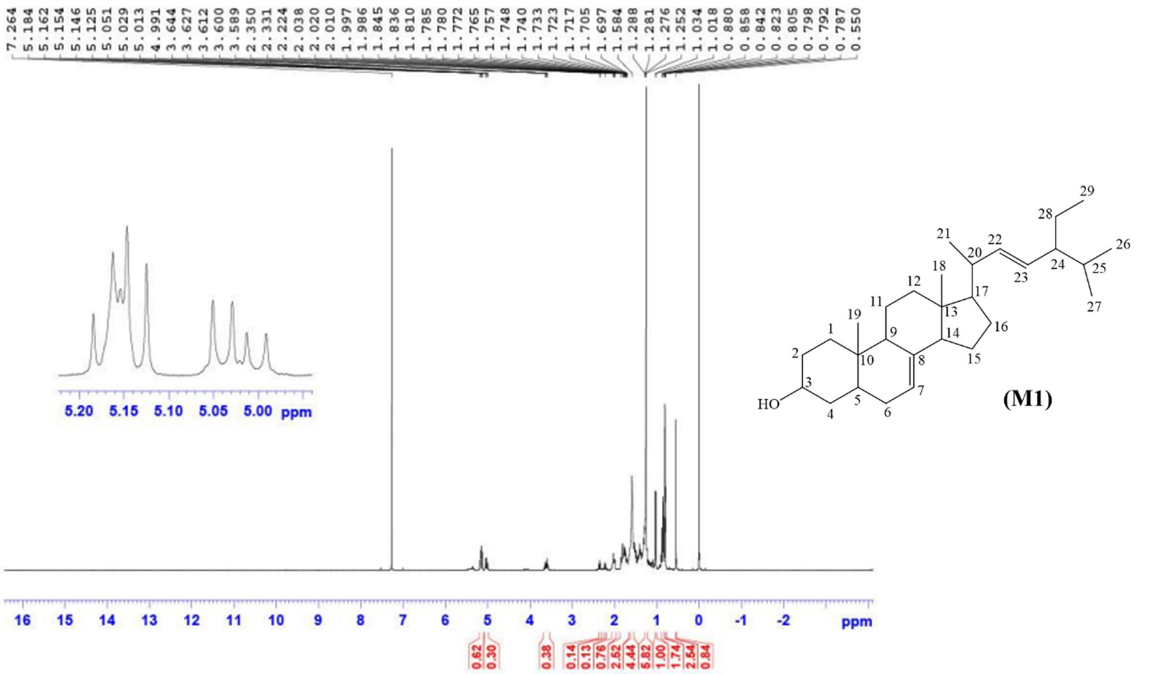

Figure S3: ^{1}H-NMR of compound M1

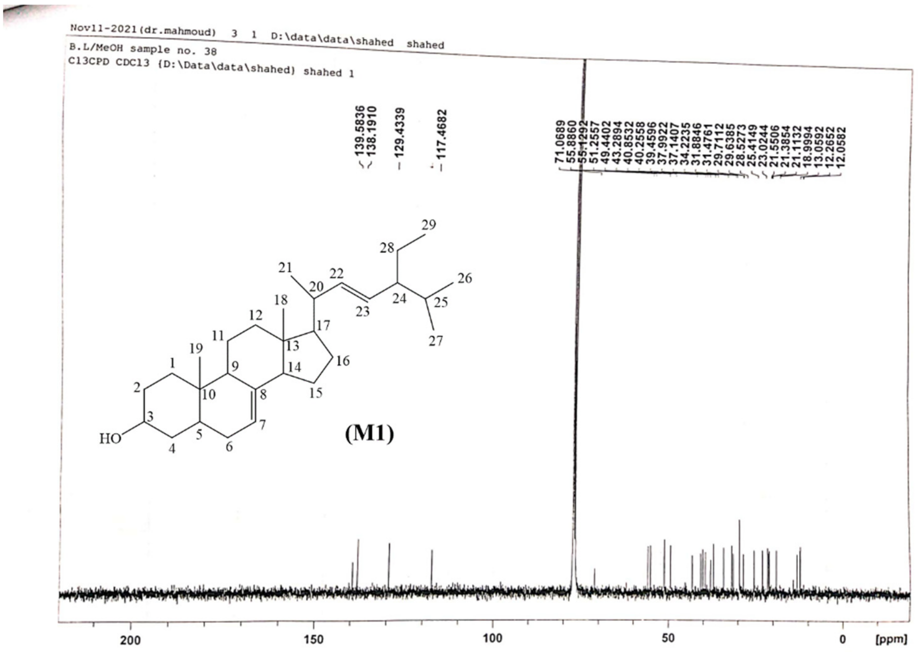

Figure S4: ^{13}C-NMR of compound M1

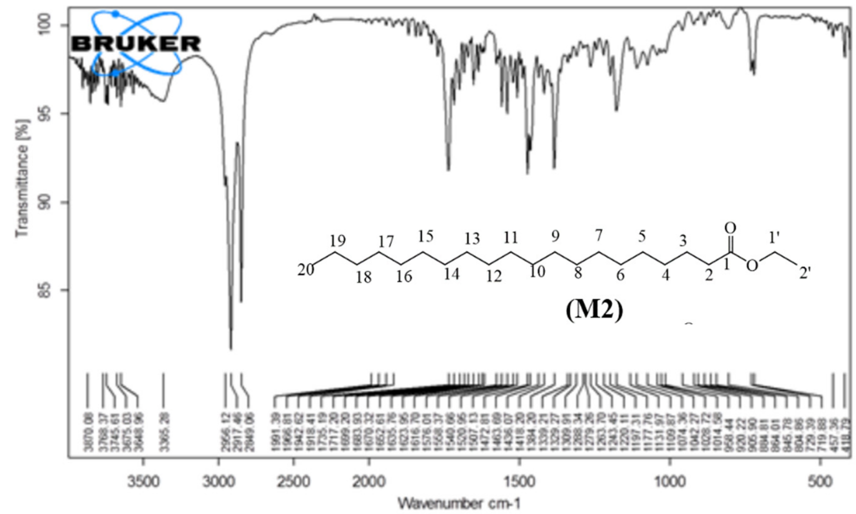

Figure S5: IR of compound M2

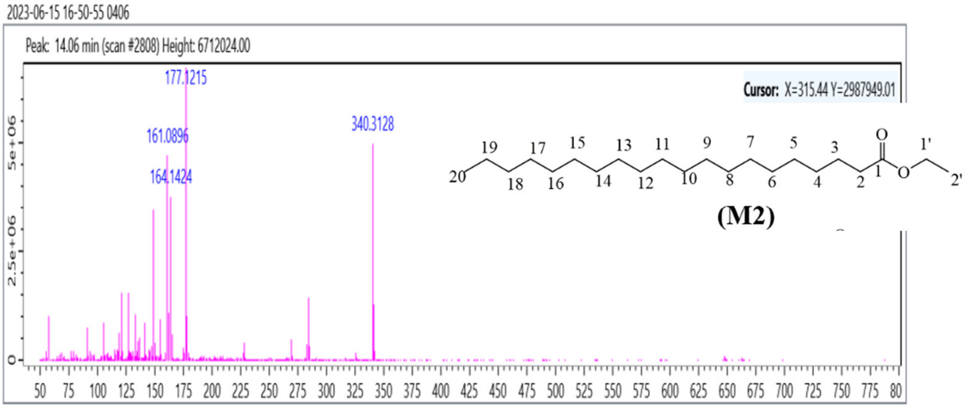

Figure S6: Mass of compound M2

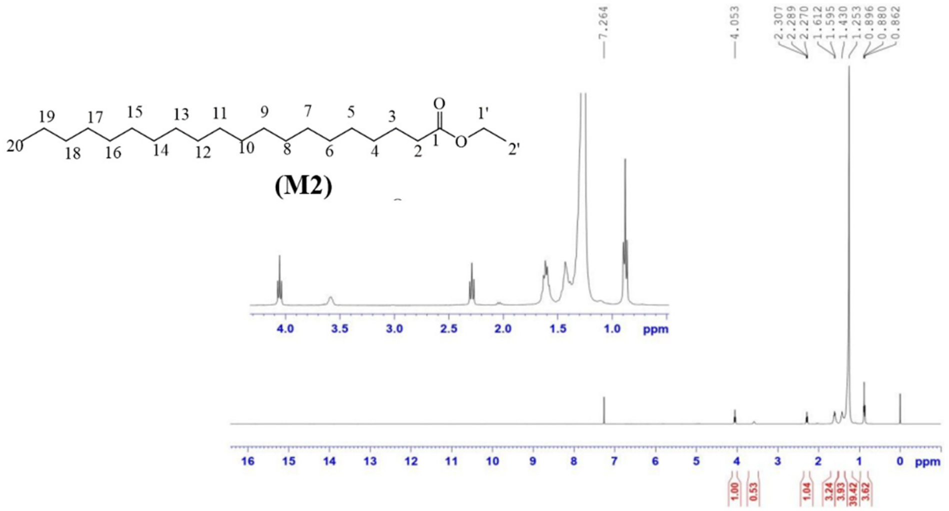

Figure S7: ^{1}H-NMR of compound M2

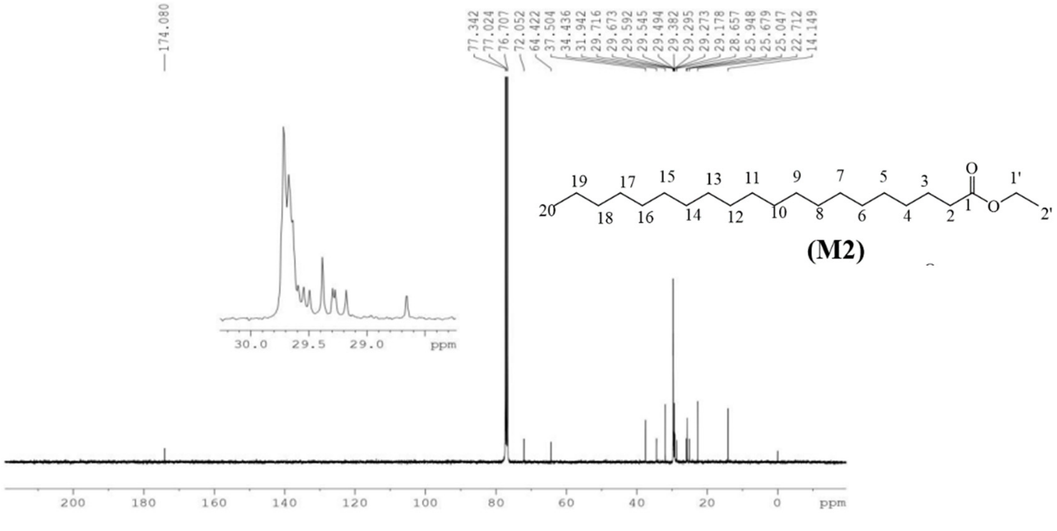

Figure S8: ^{13}C-NMR of compound M2

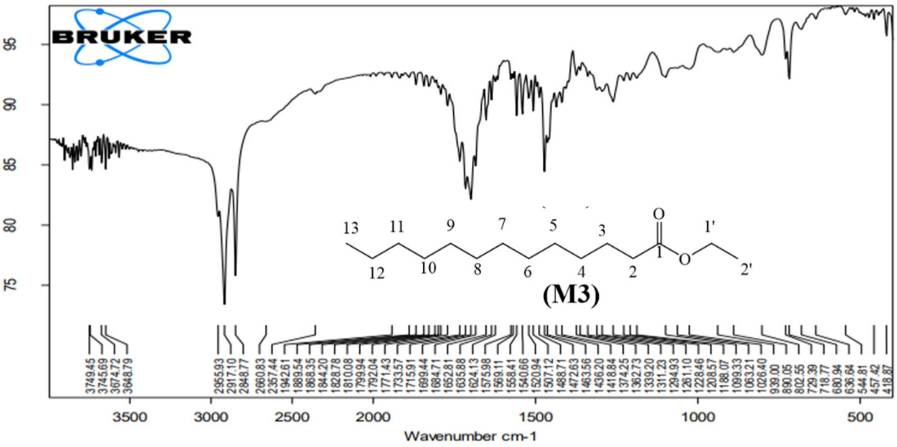

Figure S9: IR of compound M3

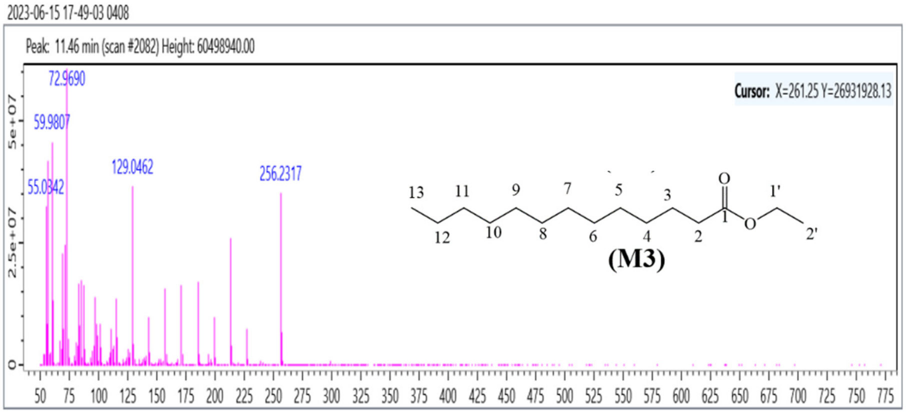

Figure S10: Mass of compound M3

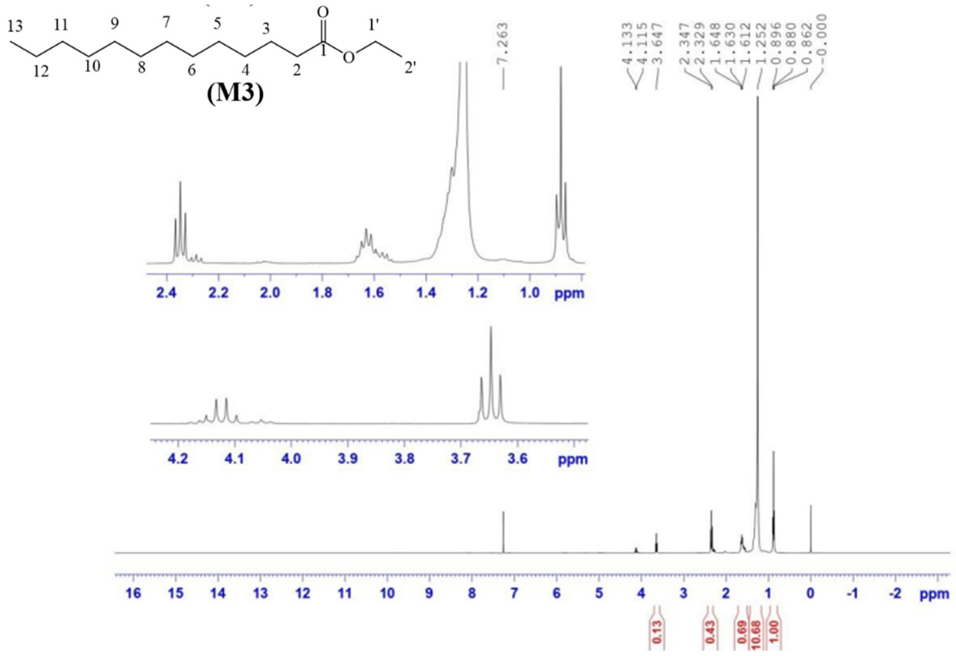

Figure S11: ^{1}H-NMR of compound M3

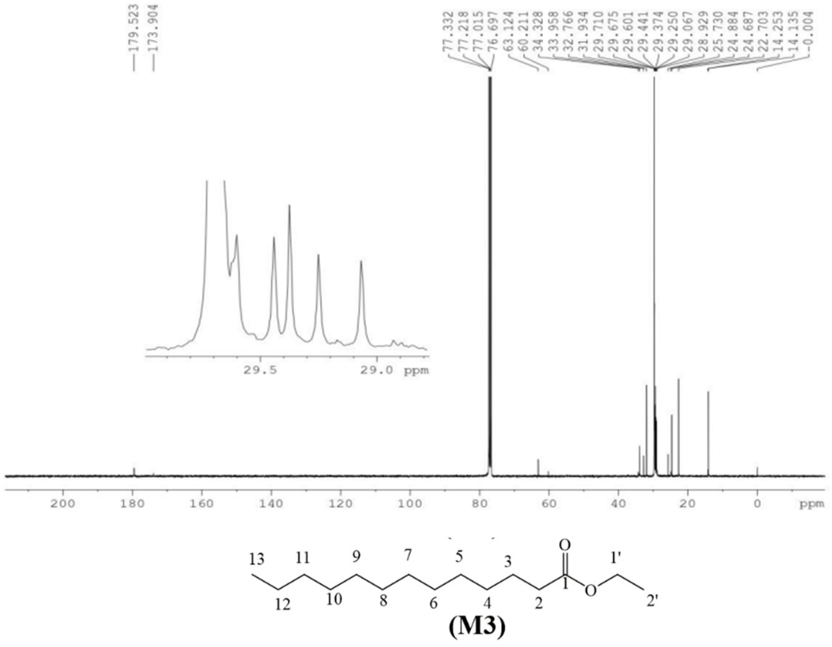

Figure S12: ^{13}C-NMR of compound M3

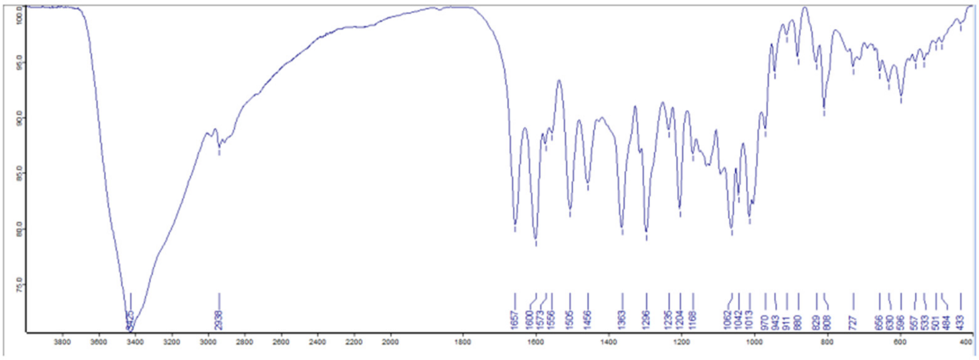

Figure S13: IR of compound B1

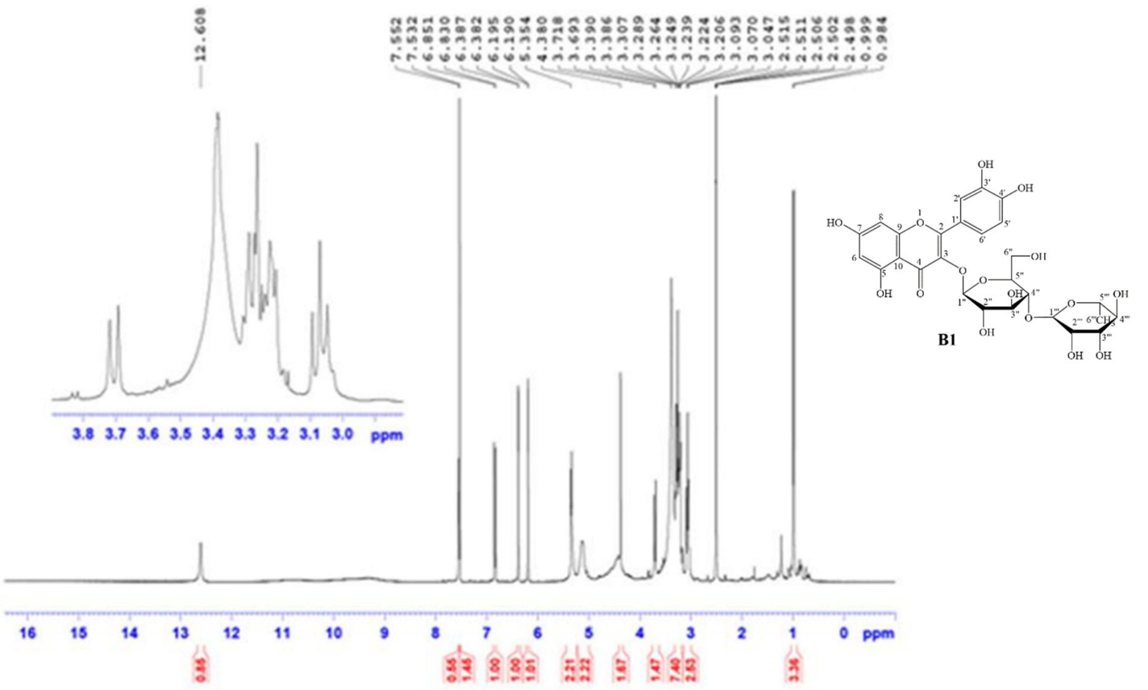

Figure S14: ^{1}H-NMR of compound B1

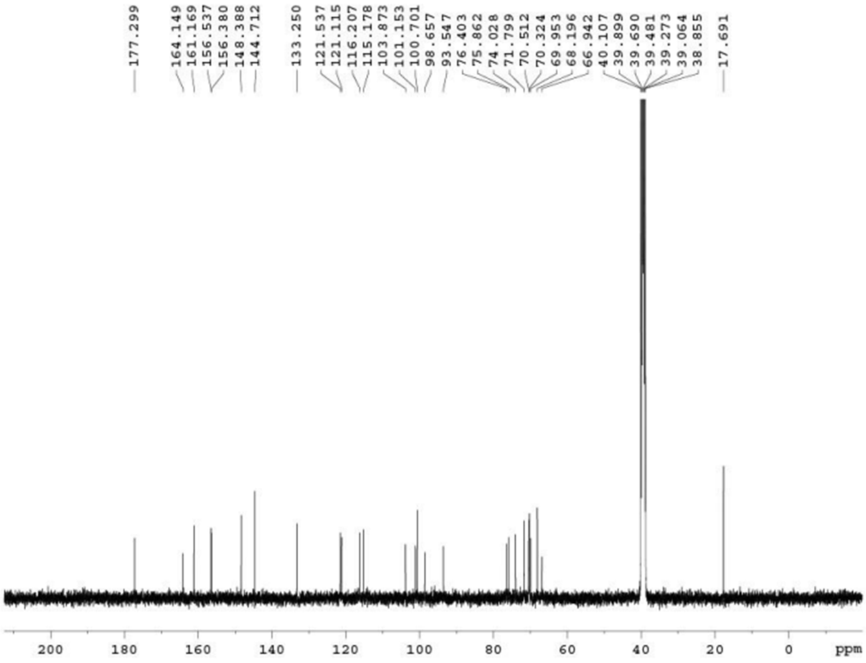

Figure S15: ^{13}C-NMR of compound B1

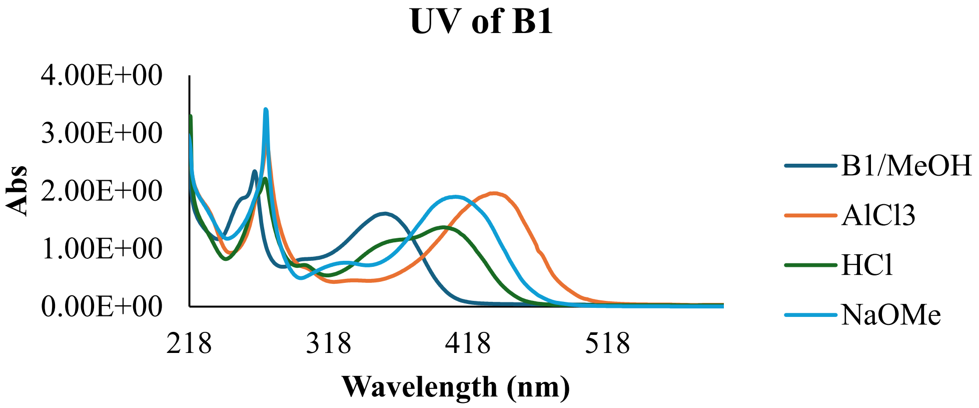

Figure S16: UVI-Vis of compound B1

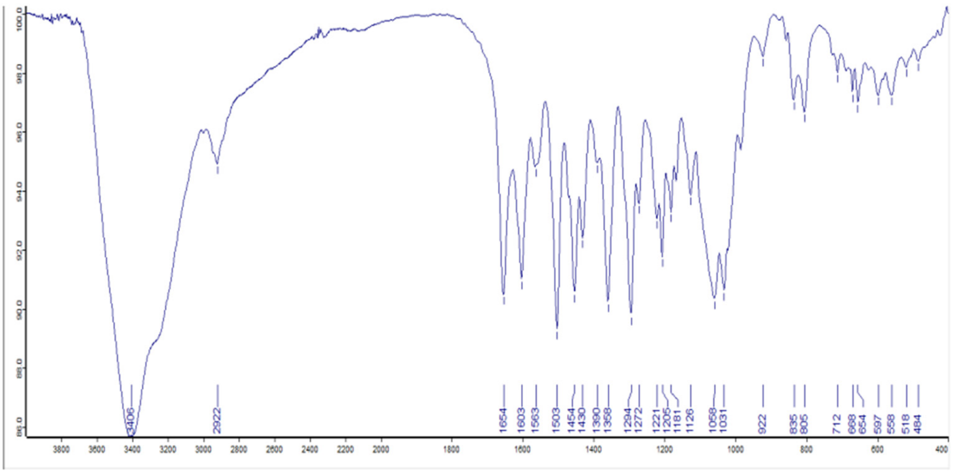

Figure S17: IR of compound B2

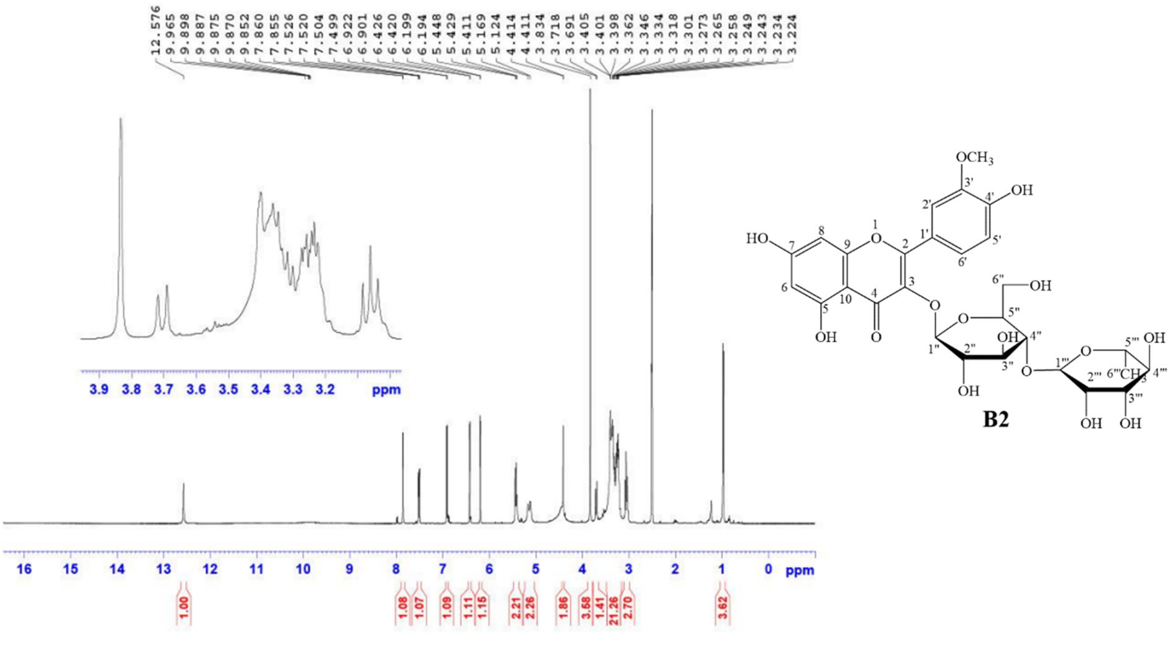

Figure S18: ^{1}H-NMR of compound B2

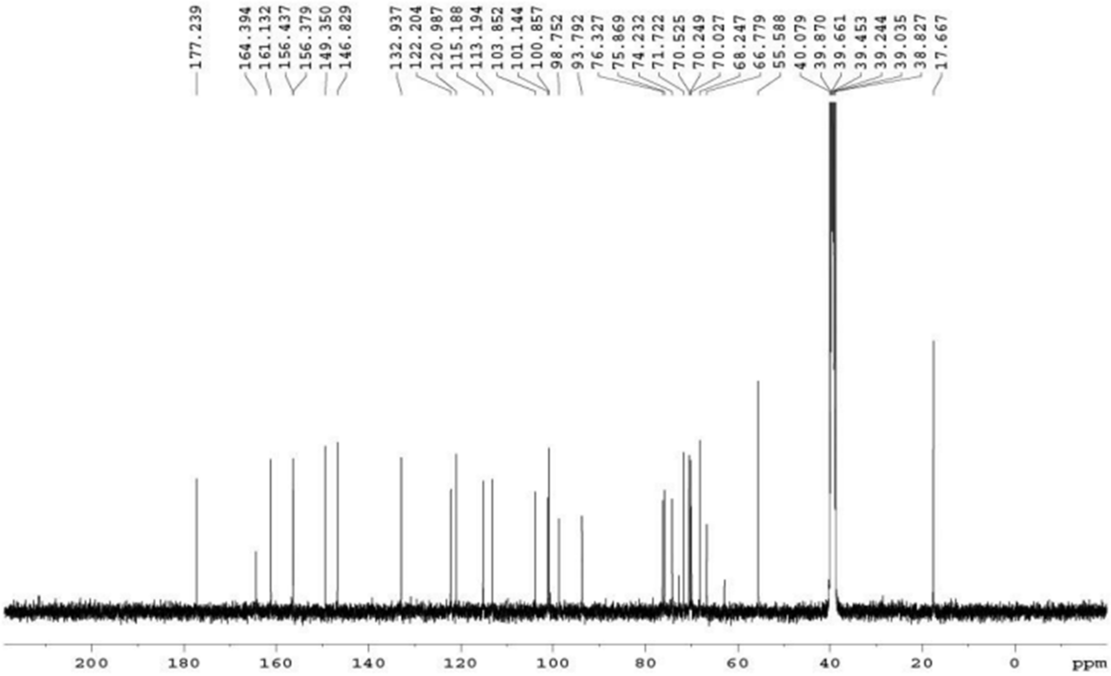

Figure S19: ^{13}C-NMR of compound B2

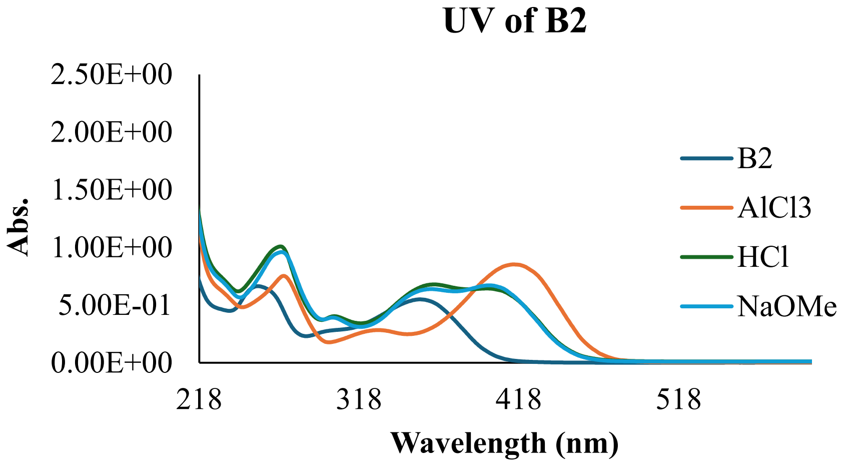

Figure S20: UV-Vis of compound B2

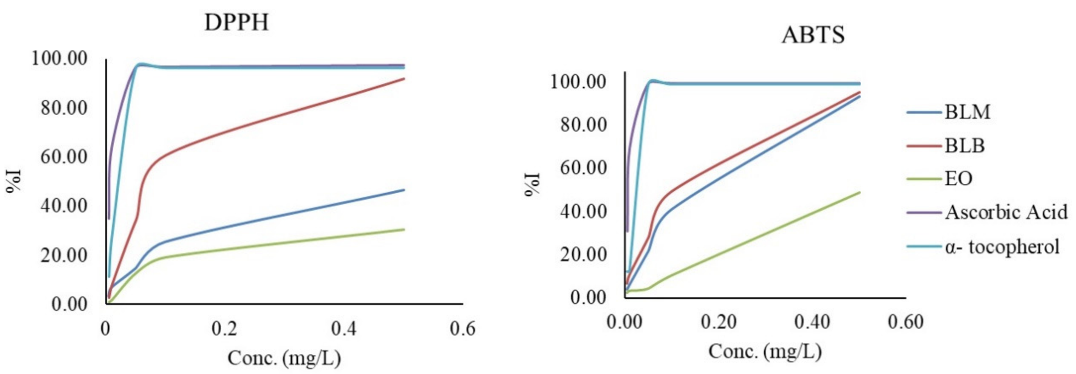

Figure S21: DPPH and ABTS radical scavenging activity results for the tested fractions and HDEO
